# The influence of behavioral context on sensory encoding

**DOI:** 10.1186/1471-2202-12-S1-P295

**Published:** 2011-07-18

**Authors:** Matthew Chalk, Iain Murray, Peggy Seriès

**Affiliations:** 1 School of Informatics, University of Edinburgh, Edinburgh, EH8 8AB, UK

## 

The properties of sensory neurons are not fixed, but change dynamically according to the task being performed [1]. While there has been a huge experimental effort put into characterizing these effects, a clear understanding of *why* they occur is lacking.

A large body of research focuses on the idea that the visual system learns a probabilistic model of natural image statistics. Typically, the goal of the visual system is seen as inferring the hidden causes underlying a given sensory input [2]. While this framework is successful in helping to understand the properties of neurons in the early sensory cortex, it presents a passive view of learning: the sensory representation is optimized independently of behavioral demands.

We propose an alternative normative framework for modeling visual processing, with the representation optimized adaptively in order to facilitate interaction with the environment [3,4]. This framework is used to ask the following questions: (1) under what conditions should behavioral context influence the responses of sensory neurons; (2) what are the expected changes in receptive field properties?

We simulate a visual detection task where an agent is presented with stimuli at various locations, and has to report whether or not a stimulus is present at a single task-relevant location. Stimuli are represented by binary latent variables (each variable corresponding to a different spatial location), which combine linearly to produce the sensory input. The task is thus to infer the state of a single task-relevant latent variable based on the sensory input: correct responses result in an immediate reward.

In performing the task, the agent is assumed to follow a strategy whereby they parameterize the expected value of state-response pairs using a probabilistic model describing how sensory data are generated from hidden causes (‘sensory encoding’), and a utility function describing the expected reward for different responses, given the hidden state (‘reward encoding’). The parameters of the model and of the utility function are learned simultaneously to fit both the distribution of inputs, and ‘desired' responses in the task (estimated using the received reward).

When there were no limitations on what could be learned, we found that the task had no influence on the learned model. Therefore, we hypothesized that task-dependent changes in sensory encoding occur due to a computational resource limitation, when there is a compromise between explaining all the sensory inputs, and enabling good task performance. Specifically, we considered the case where there are fewer hidden units in the learned model than in the true model generating the data.

In this condition, we found that the task strongly affected the learned model, with basic functions corresponding to task-relevant hidden units achieving the closest fit to the true model. Significantly, task-relevant hidden units showed increased activation in response to preferred stimuli, compared to task-irrelevant hidden units (due to the reduction in uncertainty associated with learning a better model), analogous to the experimental effects of attention, found in low to mid-level areas of the visual cortex [1]. Finally, we tested the ability of the model to account for other observed effects of attention, such as multiplicative scaling of neuronal tuning curves, biased competition and modulation of centre-surround interactions [1].
